# Edge-Oriented Graphene on Carbon Nanofiber for High-Frequency Supercapacitors

**DOI:** 10.1007/s40820-017-0162-4

**Published:** 2017-10-26

**Authors:** Nazifah Islam, Juliusz Warzywoda, Zhaoyang Fan

**Affiliations:** 1 Department of Electrical and Computer Engineering and Nano Tech Center, Texas Tech University, Lubbock, TX 79409 USA; 2 Materials Characterization Center, Whitacre College of Engineering, Texas Tech University, Lubbock, TX 79409 USA

**Keywords:** High-frequency supercapacitor, Kilohertz supercapacitor, Vertical graphene, Carbon nanofiber, AC filtering

## Abstract

High-frequency supercapacitors are being studied with the aim to replace the bulky electrolytic capacitors for current ripple filtering and other functions used in power systems. Here, 3D edge-oriented graphene (EOG) was grown encircling carbon nanofiber (CNF) framework to form a highly conductive electrode with a large surface area. Such EOG/CNF electrodes were tested in aqueous and organic electrolytes for high-frequency supercapacitor development. For the aqueous and the organic cell, the characteristic frequency at − 45° phase angle was found to be as high as 22 and 8.5 kHz, respectively. At 120 Hz, the electrode capacitance density was 0.37 and 0.16 mF cm^−2^ for the two cells. In particular, the 3 V high-frequency organic cell was successfully tested as filtering capacitor used in AC/DC converter, suggesting the promising potential of this technology for compact power supply design and other applications.

**Electronic supplementary material:**

The online version of this article (doi:10.1007/s40820-017-0162-4) contains supplementary material, which is available to authorized users.

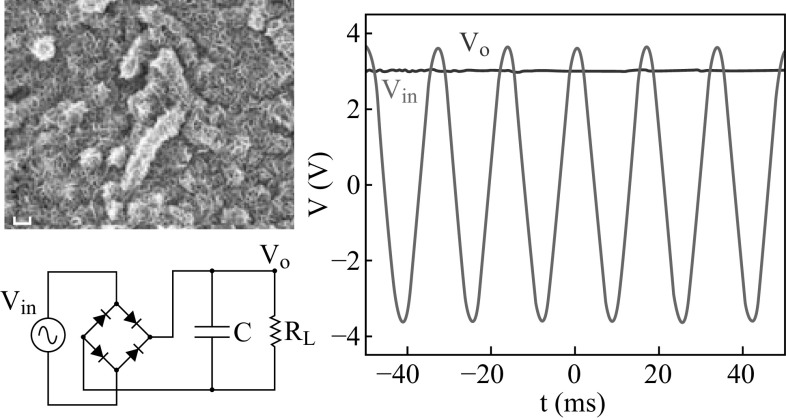

## Highlights

- 3D edge-oriented graphene (EOG) was grown encircling carbon nanofiber (CNF) framework to form a highly conductive electrode with a large surface area.
- EOG/CNF-based supercapacitors in both aqueous and organic electrolytes can response at the kilohertz frequency.
- 3 V high-frequency organic supercapacitor was tested as filtering capacitor used in an AC/DC converter.

## Introduction

Great efforts are being devoted to developing supercapacitors with large energy density to bridge the performance gap between supercapacitors and batteries [1–11]. In a second frontier, there also exists a huge gray area on the Ragone plot between supercapacitors and electrolytic capacitors. Supercapacitors provide much larger capacitance than electrolytic capacitors, but their frequency response is several orders of magnitude lower, typically limited below 1 Hz, whereas the latter can response at kilohertz (kHz) or higher. Therefore, conventional supercapacitors cannot work as capacitors for current ripple filtering and pulse smoothing. Developing high-frequency supercapacitors that can respond at kHz frequencies [12, 13] is an interesting area that will find many applications in power electronics, DC power design, and pulse power storage and generation, in replacing the currently used electrolytic capacitors that have a much lower capacitance density, and therefore, a bulky size.

The frequency response of an electric double layer-based supercapacitor is mainly restricted by the migration rate of electrolyte ions in the porous electrode and the electronic conductivity of the electrode itself [14, 15]. Using electrode materials with high conductivity and large pores is therefore crucial to develop high-frequency supercapacitors. To this end, vertically oriented graphene (VOG) on 2D substrates [12, 16, 17], edge-oriented graphene (EOG) in 3D foam [18, 19], sub-micrometer ultrathin carbon nanotube (CNT) films [20], well-connected carbon nanofiber aerogel [21] and others [22–27] have been reported as electrodes of high-frequency supercapacitors.

Here, we report kHz supercapacitors with AC filtering capability, which are based on EOG grown on carbon nanofibers (CNF) scaffolds. 3D EOG was grown encircling individual CNFs to form a highly conductive electrode with a large surface area. Such EOG/CNF electrodes were studied in aqueous and organic electrolytes to confirm their kHz high-frequency response and large capacitance. Organic supercapacitor cells that can run up to 3 V were also tested for 60 Hz line-frequency filtering in an AC/DC converter to achieve steady state 3 V D output, thus verifying the promising potential of high-frequency supercapacitors in substitution of electrolytic capacitors for current ripple filtering and pulse smoothing.

## Experimental Section

### CNF and EOG/CNF Films Preparation and Structural Characterization

One gram of commercial CNF (Sigma-Aldrich) was mixed with 0.5 g ethyl cellulose, 0.1 g lauric acid and 6 mL terpineol by grinding finely in a mortar. This thick paste was diluted with anhydrous ethanol. Finally, few drops of surfactant (Triton-X 100) were added to prevent agglomeration of nanofibers. The mixture was sonicated in an ultrasonic bath for several hours to obtain a uniform dispersion. The obtained paste was coated on a thin Ni foil by spin coating at a low speed. The coated substrate was dried in air, followed by heating at 300 °C for 30 min in an oven to evaporate the residual organic components. The dried CNF-coated Ni foil was then cut into pieces for EOG growth. The detailed procedure can be found in Ref. [28]. Briefly, EOG growth was conducted in a PECVD reactor underflow of hydrogen (100 sccm) and methane (50 sccm) gases. The pressure was set at 30 Torr. The stage was heated to 750 °C before starting plasma. The microwave plasma was turned on with a power of 1000 W, and the growth time was 7.5 min.

Microscopic imaging for analyzing the morphology and crystalline structure of CNF and EOG/CNF films were done by using LEO SUPRA 35 scanning electron microscope and Hitachi H-9500 transmission electron microscope. Raman spectroscopic studies of CNF and EOG were carried out using a microRaman system (Bruker SENTERRA) with an excitation laser of 532 nm.

### Electrochemical Test

For electrochemical tests in aqueous and organic electrolytes, 6 M KOH and 1 M TEABF_4_ in anhydrous acetonitrile (AN) solutions were used, respectively. For aqueous cells, a filter paper was used as a separator and was soaked in electrolyte overnight. The wet paper was placed between the two electrodes, and the assembly was pressed together for the test. The separator used for organic electrolyte was commercial Celgard 3501. Organic electrolyte-based coin cells were assembled in an Ar-filled glove box with water and oxygen content less than 0.1 ppm. Electrochemical measurements were conducted using a Biologic SP 150 electrochemical workstation. Electrochemical impedance spectroscopy (EIS) was measured from 100 kHz to 1 Hz with a sinusoidal AC voltage of 10 mV amplitude. Cyclic voltammetry (CV) was carried out in 0–0.9 V range for KOH and in 0–2.5 V range for organic electrolyte at different scan rates from 1 to 600 V s^−1^.

## Results and Discussion

**Fig. 1:**
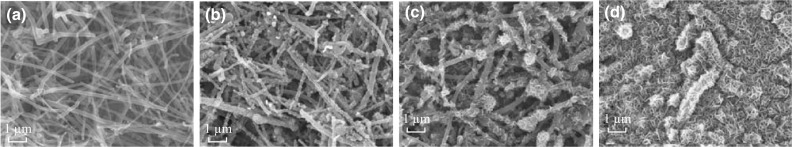
SEM images of **a** pure CNF thin film, and EOG/CNF films for EOG growth time of **b** 5 min, **c** 7.5 min, and **d** 10 min

SEM images of CNF and EOG/CNF layers are presented in Fig. 1. Individual CNFs with a diameter between 100 and 300 nm are entangled together to form a thin layer (Fig. 1a). As shown in Fig. S1a in the supplementary information (SI), the film thickness was controlled to be around 1 µm. Figure 1b–d shows the surface morphology of EOG/CNF with EOG growth time of 5, 7.5, and 10 min, respectively. The growth mechanism of graphene was explained in previous studies [29, 30]. Briefly, the growth process can be divided into three major steps. At the very moment of plasma ignition, free carbon radicals from carbon precursors are deposited on the substrate to form a graphitic layer, which, due to certain mechanical and thermal factors, ends up with vertically bent cracks. This basal layer, being graphitic with dominant *sp* ^2^ hybridized carbons, provides a highly conductive interface between the substrate and the vertical graphene sheets. In the second step, few-layer graphene starts growing from these cracks along the vertical direction. The electric field in the plasma guides the bombardment of carbon atoms preferably along the vertical direction, hence forming vertically oriented graphene. The third step is the growth of graphene sheets in vertical direction as long as the plasma is active. In our case, the thin carbon fiber mesh acts as the substrate. Since graphene sheets grow perpendicular to CNF in all orientations with fully exposed graphene edges, they are called as edge-oriented graphene (EOG) [28].

For 5 min growth period (Fig. 1b), only sparse EOG flakes were observed, which do not provide enough surface area. However, when the growth time was increased to 10 min (Fig. 1d), highly dense EOG flakes were deposited, blocking the macropores that are necessary to facilitate rapid ion transport for high-frequency response. The perpendicular orientations of 3D EOG flakes that encircle each CNF can be clearly observed with graphene edges exposed. We selected a growth time of 7.5 min in this study (Fig. 1c). Obtained after this growth time, relatively uniform EOG covers CNF to achieve a large surface area, while macropores are still retained, which can promote electrolyte diffusion. The deposited EOG also acts as a mechanical and electrical “glue” to provide a good connection between individual CNFs by reducing their contact resistance, thus enhancing the conductivity of the whole electrode structure. This is crucial for high-frequency response (Fig. S1b).

**Fig. 2:**
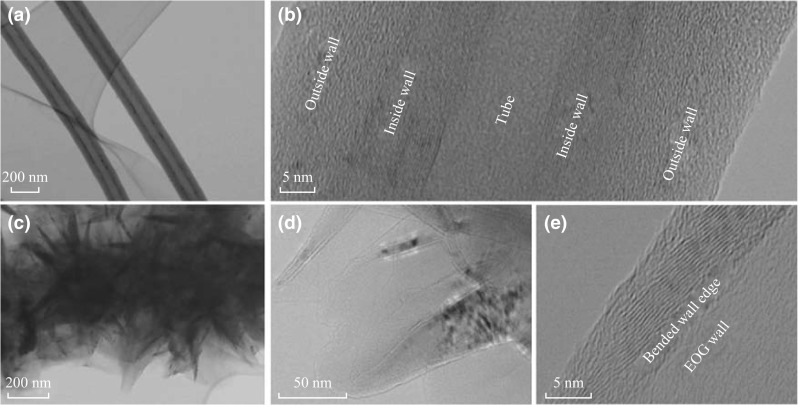
**a** TEM image of CNF showing its tubular structure. **b** High-resolution TEM image of the tubular CNF. **c** TEM image of the EOG flake clusters. **d** TEM image of individual EOG flakes showing the bent flake morphology. **e** High-resolution TEM image focused on the sidewall edge of one bent EOG flake showing the multiple graphene layers

Detailed microscopic characterization of CNF and EOG was obtained from TEM study. Individual CNFs have a tubular structure (Fig. 2a). The lattice image shown by the high-resolution TEM (Fig. 2b) indicates that the crystallization (graphitization) of CNF tube wall is not uniform. The inside wall is highly graphitized, while the outside wall is more like amorphous carbon. The cluster of multiple perpendicularly oriented graphene flakes are shown in Figs. 2c and S2a, while the orientation and morphology of individual EOG flakes can be identified in Fig. 2d. In particular, the flake is perpendicular to the CNF with graphene edge exposed. The flake wall is not flat but with a bent morphology. Therefore, when focused at the bent wall edge for imaging, the lattice of multiple graphene layers is observed (Figs. 2e and S2b). The multilayer EOG flake has a tapered profile with easily accessed and high-density graphene edges. It is known that these atomic edges or kinks have an electrochemical reactivity several orders of magnitude higher than its basal plane [31–33]. In particular, these edges, in addition to the in-plane surface, are adsorption sites of electrolyte ions, thus augmenting achievable capacitance [19].

To further investigate the material properties of edge-oriented graphene sheets grown on carbon nanofibers, Raman spectroscopy study was performed. The Raman spectrum of EOG/CNF is presented in Fig. S3, which is similar to the feature typically observed in vertical graphene structures [16, 19]. The Raman spectrum of CNF is also presented for comparison. Both CNF and EOG/CNF show distinct D, G, and 2D peaks, with D and G being located at 1350 and 1580 cm^−1^, respectively, while the 2D peak moves from 2680 to 2700 cm^−1^ after EOG growth. The peak widths for EOG are significantly smaller than those of bare CNF. The intensity ratio of D and G peaks provides information about the defect density. The *I*_D_/*I*_G_ for EOG/CNF was found to be 0.40, while for bare CNF, it is 0.79. Since the *I*_D_/*I*_G_ is inversely proportional to the average size of the *sp* ^2^ domains, the crystallization domain size in EOG is therefore larger than CNF. Besides, the *I*_2D_/*I*_G_ ratio of EOG/CNF is 0.77, indicating that the EOG sheets comprised multilayer graphene [34, 35]. This was also confirmed from the TEM observation in Fig. 2.

**Fig. 3:**
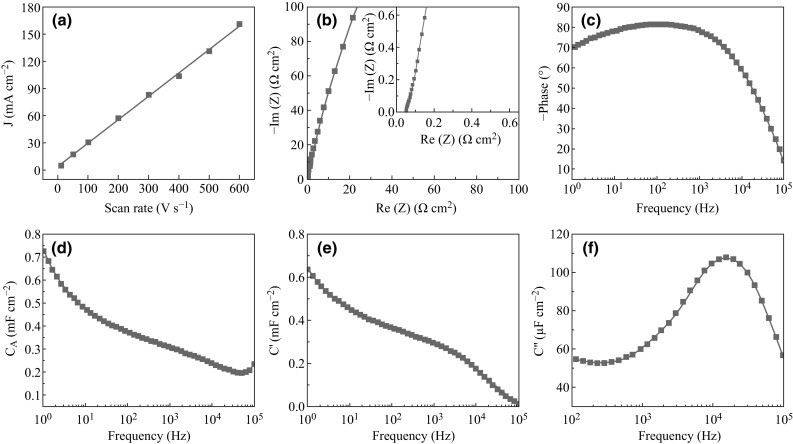
Performance study of EOG/CNF electrodes in 6 M KOH electrolyte. **a** Current density versus scan rate. **b** Nyquist complex impedance spectra. **c** The Bode phase spectrum. **d** Area specific capacitance versus frequency of a single electrode. **e** Real and **f** imaginary components of the complex capacitance

With EOG/CNF thin films as electrodes, we assembled symmetric cells using 6 M KOH aqueous electrolyte for their electrochemical performance testing. In cyclic voltammetry study, the cells were scanned at rates up to 600 V s^−1^. Several representative CV curves are presented in Fig. S4. For comparison, the CV profiles form pure CNF-based cells are also plotted. At such high scan rates, these cells maintained the desired rectangular-like response. The capacitance of EOG/CNF electrode is more than two times of that CNF electrode due to the extra surface area contributed by EOG. The current density at different scanning rates for the EOG/CNF cell is shown in Fig. 3a. It linearly increases with the increasing scanning rate, confirming the rapid response of the cell.

The frequency response of the EOG/CNF cells was characterized by EIS study, measured from 100 kHz–1 Hz. The complex plane impedance plot (Nyquist plot) is presented in Fig. 3b, with the high-frequency region shown as the inset. The equivalent series resistance (ESR) is found to be as small as 0.05 Ω cm^2^ in the aqueous cell. The impedance line intersects with the real axis almost vertically, suggesting a trivial porous effect. The frequency-dependent phase plot (Bode plot) is shown in Fig. 3c. It is observed that for frequencies between 100 and 1000 Hz, the absolute value of the phase angle is above 80°, suggesting a small resistive loss in this frequency region. In particular, at 120 Hz, the cell has a phase angles of − 81.5°. The characteristic or cutoff frequency of a capacitor is defined by the frequency at which its phase reaches − 45°, assuming a simple series RC model. Beyond this characteristic frequency, the device is considered to be more like a resistor than a capacitor. The characteristic frequency of EOG/CNF aqueous cell is found to be as large as 22 kHz. Based on the RC model, the cell capacitance can be extracted. The dependence of the electrode areal capacitance (*C*_A_) on frequency is shown in Fig. 3d. At 120 Hz, this capacitance is 0.37 mF cm^−2^. This value is larger than that reported for electrodes based on VOG grew on a flat substrate [12, 16, 36]. Moreover, compared to other reports on EOG grown on 3D scaffolds, our supercapacitor has distinctive high volumetric capacitance density. Specifically, considering the active electrode material thickness, which, for EOG/CNF layer is ~1 µm, the aqueous capacitors have a large volumetric density of 3.7 F cm^−3^, which is considerably higher than those obtained from low surface area scaffolds like Ni foam [18], owing to high specific surface area of ultrathin nanofibers.

The frequency-dependent capacitive behavior can be analyzed using another method by defining a complex capacitance [25, 37], C = C′ − *j*C″, where C′ corresponds to actual capacitance and C″ accounts for the losses due to resistance associated with capacitance. The area-specific values of C′ and C″ were calculated. In principle, C′ should be consistent with the capacitance calculated from the *RC* model in Fig. 3d. As shown in Fig. 3e, the area-specific C′ values are similar to the values in Fig. 3d, and at 120 Hz, the extracted areal capacitance is 0.36 mF cm^−2^. The peak of the C″ versus frequency (Fig. 3f) reveals the relaxation time constant, which is the inverse of the frequency at which C″ reaches its peak value. Here, the relaxation time is 0.07 ms.

Capacitive behavior and cycling stability of EOG/CNF electrodes were also tested under Galvanostatic condition. Applying a steady current of 40 mA cm^−2^, the aqueous capacitor was cycled for 1 million times. The capacitive property was found extraordinarily satisfactory as the retention remained within 98%–100%. Moreover, complex impedance was measured before cycling and after completion of 1 million cycles. The impedance curves were found to have a negligible difference, with the ESR remaining the same as initial (Fig. S8). Such outstanding stability proves the chemical and mechanical robustness of the EOG/CNF composite film.

**Fig. 4:**
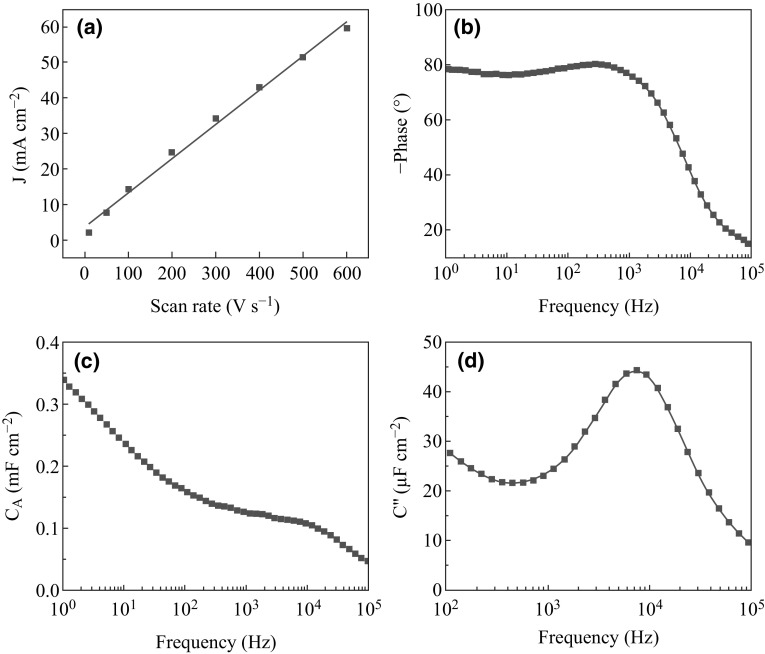
**a** Current density versus CV scan rate of EOG/CNF electrodes in 1 M TEABF_4_ in acetonitrile. **b** The Bode phase spectrum of the cell. **c** Area specific capacitance versus frequency of the electrode. **d** Imaginary component of complex capacitance (*C*″) versus frequency

The potential window of aqueous electrolyte-based supercapacitors is constrained by the water-splitting potential and is usually below 0.9 V. The allowable operating voltage range is much wider in an organic electrolyte and therefore a much higher energy density. For this purpose, organic cells with 1 M TEABF_4_ in anhydrous AN solution were also studied. The CV scans were performed up to 2.5 V with scan rates to 600 V s^−1^, as shown in Fig. S5. The current densities at different scan rates are presented in Fig. 4a, which shows a linear relationship between the current densities and the scan rate, indicating desired capacitive behavior even at such high scan rates. From the Nyquist plot of the complex impedance (Fig. S6a), an ESR of 0.28 Ω cm^2^ in the organic electrolyte was found. The larger ESR for organic cells than for aqueous cells is mainly caused by the smaller ionic conductivity of organic electrolytes. This will also result in a slower frequency response. Based on the Bode plot in Fig. 4b, the cell exhibits a 120 Hz phase angle of −80°, suggesting a reasonable small loss at this high frequency. The characteristic frequency when its phase reaches −45° is 8.5 kHz. Using the series RC model, the cell capacitance can be calculated and is plotted in Fig. 4c. At 120 Hz, this capacitance is 0.16 mF cm^−2^. From Fig. 4d, the relaxation time is found to be around 0.12 ms.

The specific capacitance in an organic electrolyte is found to be lower than that in an aqueous electrolyte. The relative dielectric constants of the corresponding solvents surely have a role in this phenomenon. The electric double layer is formed on the electrode surfaces with a sub-nm scale ultrathin layer of solvent as a dielectric, separating the two layers of charges [38]. Water has a relative dielectric constant of 80.1, which is much higher than that of acetonitrile (37.5), resulting a larger capacitance in aqueous cells. The electrolyte ion size might also contribute to this capacitance difference [39].

**Fig. 5:**
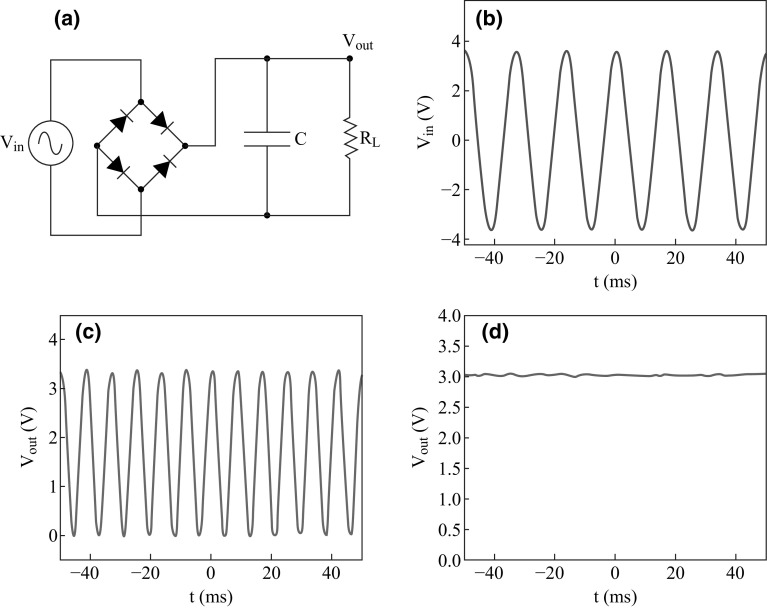
Line-frequency AC filtering demonstration of the EOG/CNF-based high-frequency supercapacitors. **a** The AC/DC conversion circuit diagram. **b** The input AC voltage at 60 Hz. **c** The full-wave rectified output in the absence of the capacitor, and **d** the DC output with the EOG/CNF supercapacitor as filtering capacitor

Finally, in order to demonstrate the practical effectiveness of these EOG/CNF-based high-frequency supercapacitors in current ripple smoothing, the assembled organic cell was tested as the filtering capacitor in the AC/DC conversion circuit as shown in Fig. 5a, with a 1 MΩ resistor as the load. The 60 Hz AC input voltage, the full-wave rectified signal in absence of the filtering capacitor, and the final DC output with the filtering capacitor are presented in Fig. 5b–d. A relative constant 3 V DC output was obtained after rectifying and filtering. This test proves the ability of these EOG/CNF-based high-frequency supercapacitors for 120 Hz filtering, in replacing bulky aluminum electrolyte capacitors, for compact power supply design. The aqueous capacitor can also be used for similar AC/DC conversion, however, with a smaller potential window (Fig. S9).

## Conclusion

3D EOG was grown encircling individual CNFs in a PECVD process to form a well-connected porous layer with a reasonably large surface area. Using such EOG/CNF film as electrodes, high-frequency supercapacitors were demonstrated in both aqueous and organic electrolytes with large capacitance. In particular, aqueous cells show a characteristic frequency of 22 kHz, 120 Hz capacitance of 0.37 mF cm^−2^, and phase angle of −81.5°. Organic cells exhibit a characteristic frequency of 8.5 kHz, 120 Hz capacitance of 0.16 mF cm^−2^, and phase angle of −80°, but can run at a voltage of 3 V. Their 120 Hz current filtering capability was further demonstrated in a 3 V AC/DC converter, indicating the promising potential of these high-frequency supercapacitors in replacing electrolytic capacitors for current filtering, pulse smoothing, and other applications.

## Electronic supplementary material

Below is the link to the electronic supplementary material.

Supplementary material 1 (PDF 1047 kb)
