# Supplementary material for: Edge-Oriented Graphene on Carbon Nanofiber for High-Frequency Supercapacitors
Source: Nanomicro Lett. 2017 Oct 26;10(1):9. doi: 10.1007/s40820-017-0162-4 (PMC6199059; doi:10.1007/s40820-017-0162-4)
Supplement: Supplementary file 1 — Supplementary material 1 (PDF 1047 kb) [file 40820_2017_162_MOESM1_ESM.pdf]

Supporting Information for

Edge-Oriented Graphene on Carbon Nanofiber for High-Frequency Supercapacitors

Nazifah Islam[1], Juliusz Warzywoda[2], Zhaoyang Fan[1, *]

[1]Department of Electrical and Computer Engineering and Nano Tech Center, Texas Tech University, Lubbock, TX 79409, USA

[2]Materials Characterization Center, Whitacre College of Engineering, Texas Tech University, Lubbock, TX 79409, USA

*Corresponding author. E-mail: Zhaoyang.Fan@ttu.edu

1 Calculation of Capacitance Density

The capacitances of symmetric cells were calculated using impedance spectroscopic data and a simple series RC circuit assumption,

$$C(f) = \frac{-1}{2\pi f Z''(f)} \qquad \text{(S1)}$$

where f is frequency and $Z''$is the imaginary part of the impedance, extracted from the impedance spectroscopy. As the electrodes are symmetric, in terms of area and volume, the area specific capacitances of the electrodes are calculated as,

$$C_A(f) = 2C(f)/A \qquad \text{(S2)}$$

with A being the area of the electrode.

The frequency dependent capacitive behavior was also analyzed by defining the complex capacitance as described in Ref. [1],

$$C = C' - jC'' \qquad \text{(S3)}$$

$$C' = \frac{-Z''}{2\pi f |Z|^2} \qquad \text{(S4)}$$

$$C'' = \frac{Z'}{2\pi f |Z|^2} \qquad \text{(S5)}$$

Where C' corresponds to the actual capacitance value and C'' represents the losses due to associated resistance. The area specific values of C' and C'' were further calculated for a single electrode as

$$C'_A = 2C'(f)/A \quad (S6)$$

$$C''_A = 2C''(f)/A \quad (S7)$$

Supercapacitive performances were analyzed by cycling voltammetry and Galvanostatic charge discharge cycling. From cyclic voltammetry data, electrode capacitances were calculated as integral of the area under the discharge CV curve being divided by scan rate, electrode area and scan voltage range,

$$C_A = 2\int_0^V J dv / (V \cdot s \cdot A) \quad (S8)$$

where J is the current density, V is the voltage range and s is the scan rate.

2 Figures and Tables

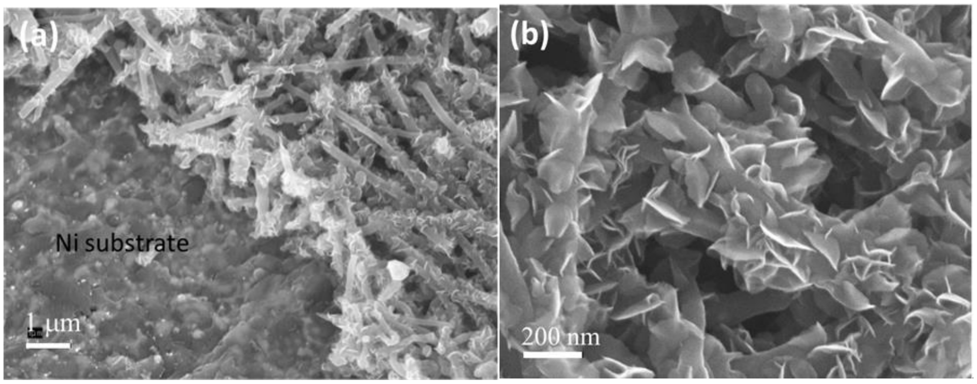

Fig. S1 SEM images of EOG/CNF film. **a** Tilted image shows that the film thickness is around 1 μm. **b** EOG flakes encircle individual CNFs and also intimately connect CNFs together

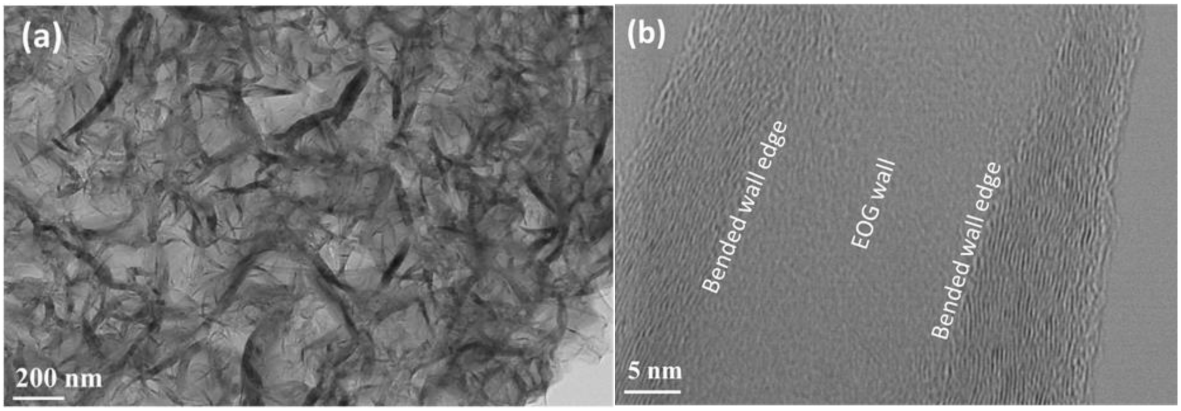

Fig. S2 TEM images of **a** EOG clusters, and **b** individual EOG flake showing the two folded sidewall edges

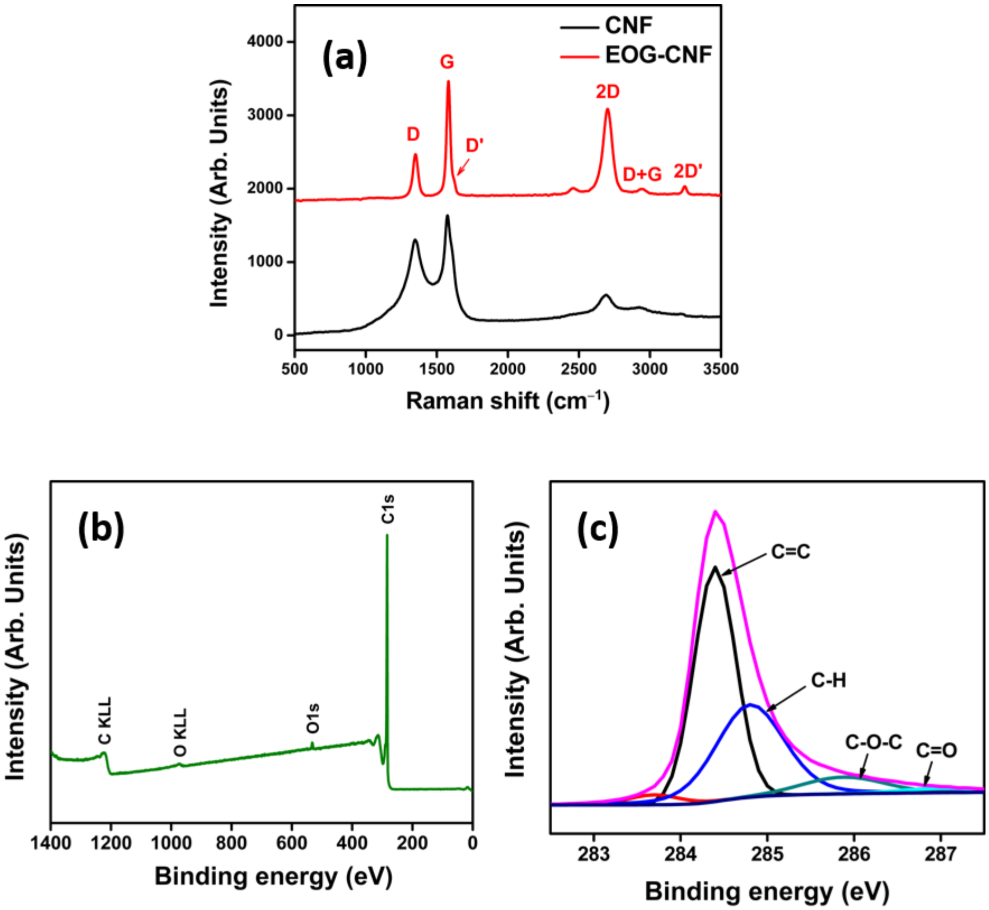

Fig. S3 **a** Raman spectra for CNF and EOG/CNF. **b** XPS survey spectrum of EOG/CNF. **c** De-convoluted C1s spectrum of EOG/CNF showing dominant C=C *sp*^{2} bonds

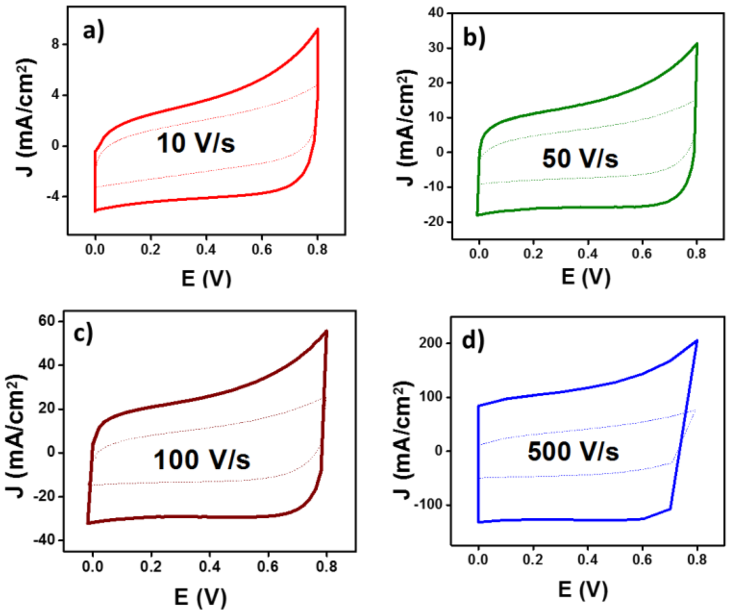

Fig. S4 Representative cyclic voltammetry curves for EOG/CNF aqueous cells at scan rates up to 500 V s^{-1}. The dotted lines are for electrodes of bare CNF

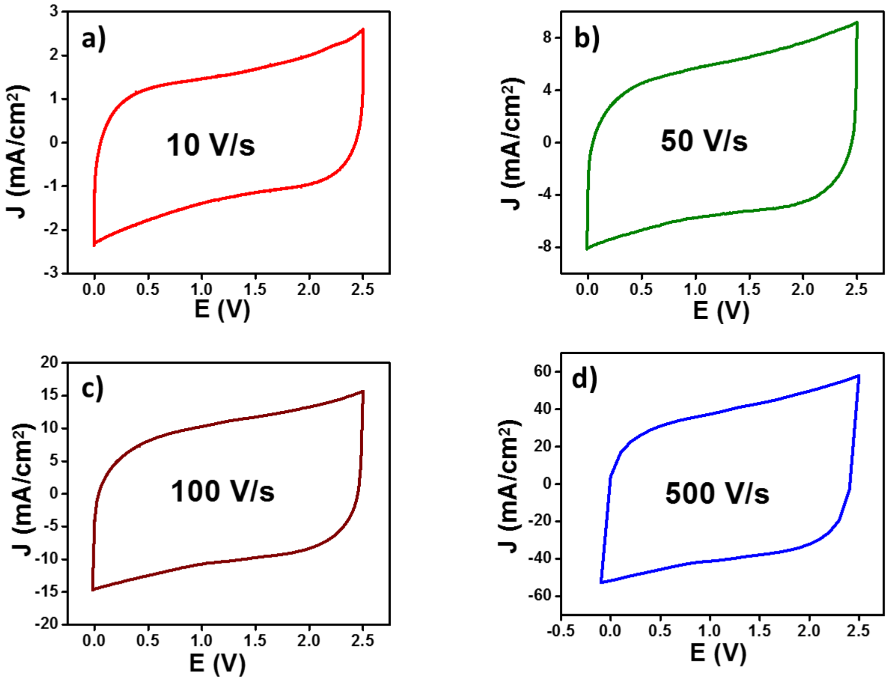

Fig. S5 Representative cyclic voltammetry curves for EOG/CNF organic cells at scan rates up to 500 V s^{-1}

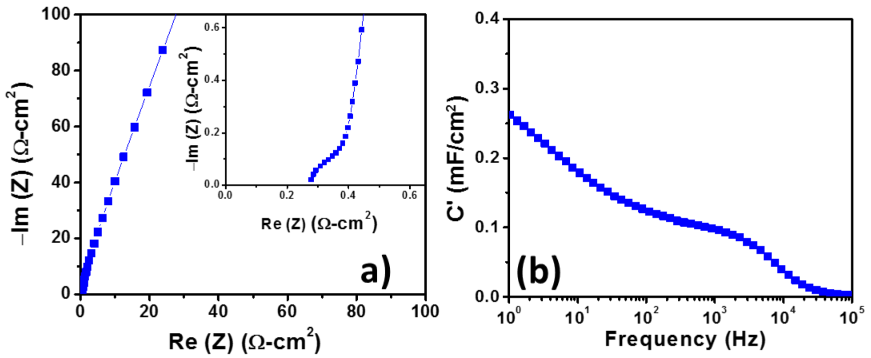

Fig. S6 a Nyquist impedance plot of EOG/CNF electrodes in organic electrolyte. **b** Real component of complex capacitance versus frequency

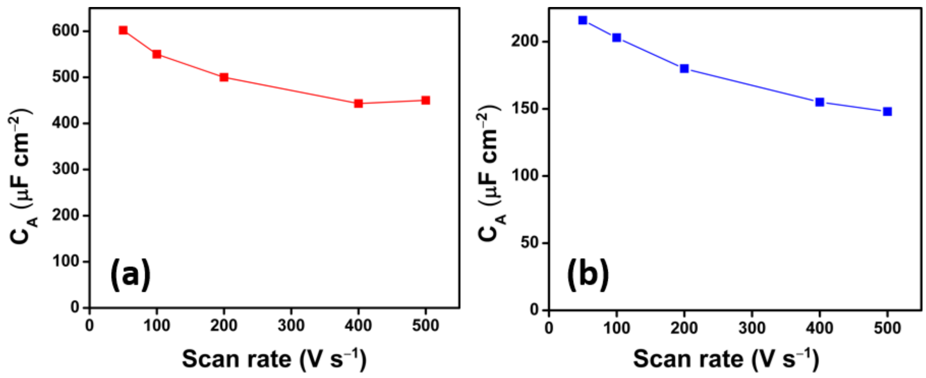

Fig. S7 Capacitance density versus scan rate for EOG-CNF capacitors in **a** aqueous electrolyte and **b** in organic electrolyte

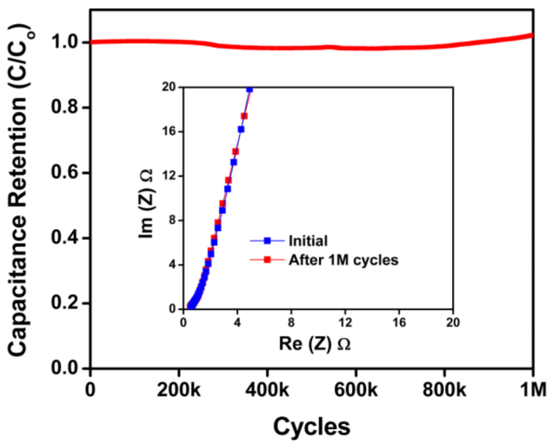

Fig. S8 Long cycle life test with aqueous capacitor at a current density of 40 mA cm^{-2}

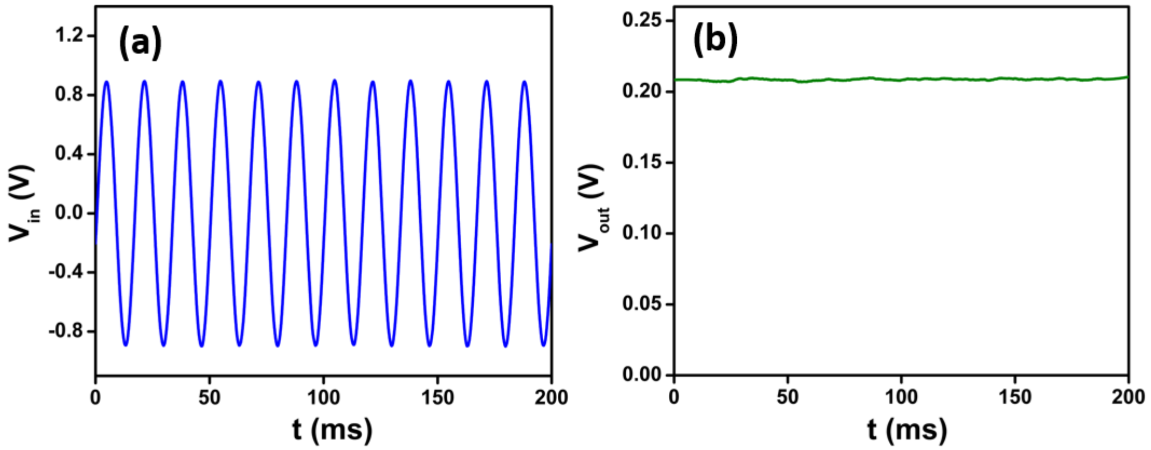

Fig. S9 AC/DC conversion with aqueous capacitor: **a** 900 mV 60 Hz sine wave input. **b** Smoothed DC output. The losses are several hundred millivolts (similar as Fig. 5) which results into very small output voltage across the capacitor

Table S1 Comparison of our results with EOG and CNT based capacitors

Electrode	f_O (kHz)	−Phase (°)	C_{A_120HZ} (mF cm^{-2})	C_{V_120HZ} (F cm^{-3})	ESR (Ω cm^2)
**VOG/Ni [2]**	15	82	0.175	0.73	0.1
**VOG/Ni [3]**	20	85	0.53		0.1
**POG/Ni foam [4]**	4	82	0.72	0.036	0.12
**EOG/CCP [5]**	12 (1 layer)	83	0.6	0.6	0.04
	5.6 (3 layers)	83	1.5	0.5	0.04
**CNT/Au/SS [6]**	1.425	81	1.202	--	0.11
**ErGO/Au [7]**	4.2	84	0.566	0.07	0.14
**EOG/CNF**	***22 (aqueous)***	***81.5***	***0.37***	***3.7***	***0.05***
**(this work)**	***8.5 (organic)***	***80***	***0.16***	***1.6***	***0.28***

References

[1] P.L. Taberna, P. Simon, J.F. Fauvarque, Electrochemical Characteristics and Impedance Spectroscopy Studies of Carbon-Carbon Supercapacitors. J. Electrochem. Soc. **150**(3), A292 (2003). doi:10.1149/1.1543948

[2] J.R. Miller, R.A. Outlaw, B.C. Holloway, Graphene double-layer capacitor with ac line-filtering performance. Science **329**(5999), 1637-1639 (2010). doi:10.1126/science.1194372

[3] M. Cai, R.A. Outlaw, R.A. Quinlan, D. Premathilake, S.M. Butler, J.R. Miller, Fast response, vertically oriented graphene nanosheet electric double layer capacitors synthesized from C(2)H(2). ACS Nano **8**(6), 5873-82 (2014). doi:10.1021/nn5009319

[4] G. Ren, X. Pan, S. Bayne, Z. Fan, Kilohertz ultrafast electrochemical supercapacitors based on perpendicularly-oriented graphene grown inside of nickel foam. Carbon **71**, 94-101 (2014). doi:10.1016/j.carbon.2014.01.017

[5] G. Ren, S. Li, Z.-X. Fan, M.N.F. Hoque, Z. Fan, Ultrahigh-rate supercapacitors with large capacitance based on edge oriented graphene coated carbonized cellulous paper as flexible freestanding electrodes. J. Power Sources **325**, 152-160 (2016). doi:10.1016/j.jpowsour.2016.06.021

[6] Y. Rangom, X.S. Tang, L.F. Nazar, Carbon nanotube-based supercapacitors with excellent ac line filtering and rate capability via improved interfacial impedance. ACS Nano **9**(7), 7248-55 (2015). doi:10.1021/acsnano.5b02075

[7] K. Sheng, Y. Sun, C. Li, W. Yuan, G. Shi, Ultrahigh-rate supercapacitors based on eletrochemically reduced graphene oxide for ac line-filtering. Sci. Rep. **2**, 247 (2012). doi:10.1038/srep00247
